# An optimized mouse parabiosis protocol for investigation of aging and rejuvenative mechanisms

**DOI:** 10.3389/fragi.2022.993658

**Published:** 2022-10-06

**Authors:** Sonia L. Rodriguez, Chase M. Carver, Andrew J. Dosch, Derek M. Huffman, Felicia D. Duke Boynton, Katayoun Ayasoufi, Marissa J. Schafer

**Affiliations:** ^1^ Department of Physiology and Biomedical Engineering Research, Mayo Clinic, Rochester, MN, United States; ^2^ Department of Molecular Pharmacology, Albert Einstein College of Medicine, Bronx, NY, United States; ^3^ Department of Medicine, Albert Einstein College of Medicine, Bronx, NY, United States; ^4^ Department of Comparative Medicine, Mayo Clinic, Rochester, MN, United States; ^5^ Department of Immunology, Mayo Clinic, Rochester, MN, United States; ^6^ Department of Neurology, Mayo Clinic, Rochester, MN, United States; ^7^ Robert and Arlene Kogod Center on Aging, Mayo Clinic, Rochester, MN, United States

**Keywords:** parabiosis, aging, rejuvenation, surgery, complications, dehiscence, blood chimerism, blood exchange

## Abstract

Surgical parabiosis enables sharing of the circulating milieu between two organisms. This powerful model presents diverse complications based on age, strain, sex, and other experimental parameters. Here, we provide an optimized parabiosis protocol for the surgical union of two mice internally at the elbow and knee joints with continuous external joining of the skin. This protocol incorporates guidance and solutions to complications that can occur, particularly in aging studies, including non-cohesive pairing, variable anesthesia sensitivity, external and internal dehiscence, dehydration, and weight loss. We also offer a straightforward method for validating postoperative blood chimerism and confirming its time course using flow cytometry. Utilization of our optimized protocol can facilitate reproducible parabiosis experimentation to dynamically explore mechanisms of aging and rejuvenation.

## Introduction

Parabiosis is the surgical pairing of organisms for the study of systemic and tissue-specific processes influenced by circulatory exchange. It enables the sharing of the circulating milieu, consisting of cells, soluble factors, and fluid, between two organisms. Each mouse serves as both recipient and donor of a unique humoral and cellular signature. Therefore, parabiosis is an intriguing model for uncovering mechanisms of both physiological and pathophysiological processes relevant to aging, regeneration, immunology, and other domains (Conboy et al., 2013; Liu et al., 2017).

**FIGURE 1:**
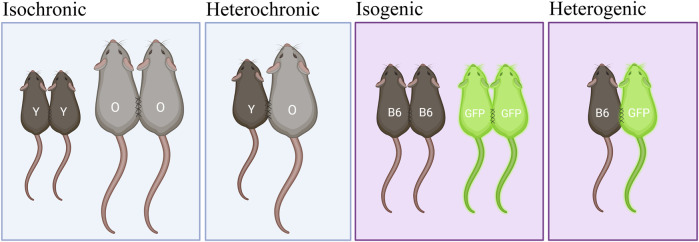
Types of surgical parabiosis pairs. Isochronic and isogenic pairs are comprised of mice of the same age or strain, respectively. Examples include young-young (YY) and old-old (OO) in an aging study and B6-B6 and GFP-GFP in a study using transgenic mice. Heterochronic and heterogenic pairs differ by age or strain, respectively. Examples include young-old (YO) in an aging study and B6-GFP in a transgenic study.

In the context of aging experimentation, heterochronic parabiosis is the surgical circulatory fusion of mice of different ages and allows mechanistic interrogation of two processes simultaneously: the effect of aged blood and systemic milieu on the young parabiont, and vice versa, the effect of young blood and systemic milieu on the aged parabiont (Figure 1). Emerging literature emphasizes the utility of the heterochronic model to reveal rejuvenative mechanisms originating from the youthful circulatory environment and conversely, progeronic mechanisms originating from the aged circulatory environment (Kiss et al., 2020; Ma et al., 2022; Pálovics et al., 2022). Heterochronic pairing must be contextualized with isochronic control pairs, comprised of same-age mice (young-young and old-old) (Figure 1), as well as young and old non-paired mice, which enable experimental standardization of age- and procedure-dependent outcomes, respectively.

Although parabiosis of rodents has been explored across the 20^th^ century (Bert, 1863; Bert, 1865; Bunster and Meyer, 1933), the modern adaptation of the model to study age-specific mechanisms is of impact and focus here. In 2005, Conboy et al. investigated heterochronic parabiosis in the context of age-related decline of regenerative potential and discovered the capacity to rejuvenate aged stem cells through exposure to young blood (Conboy et al., 2005a). In subsequent decades, parabiosis has been employed to study progeronic and rejuvenative mechanisms across organ systems. Here, we highlight seminal studies investigating the central nervous system. Villeda et al. leveraged parabiosis to identify chemokines present in aged murine plasma that contribute to brain impairments and importantly, corroborated findings with direct injection of old plasma into young mice (Villeda et al., 2011). This prompted effort to explore the converse scenario of how aged brain structure and function are modulated by exposure to a youthful circulatory environment. Ruckh et al. found that remyelination could be promoted in old mice through parabiotic exchange with young mice (Ruckh et al., 2012). Similarly, young blood stimulated dendritic spine hippocampal plasticity coinciding with improvements in learning and memory in aged mice (Villeda et al., 2014; Smith et al., 2015a). These and other studies demonstrated the dynamic utility of young and old circulatory exchange for discovery of molecules and cells that drive and respond to progeronic and youthful cues in the central nervous system (Smith et al., 2015a; Bouchard and Villeda, 2015; Smith et al., 2015b; Iram et al., 2022).

The surgical parabiosis model has revealed fundamental processes governing aging and rejuvenation with important translational impact. However, literature describing model-dependent complications and solutions are limited. The methodology described here seeks to inform practices to accomplish successful parabiotic pairing, postoperative care, and management of adverse outcomes. This parabiosis protocol describes the surgical fusion of two mice internally at the elbow and knee joints with continuous external union of the skin. It is an adaptation of the methods described by Kamran et al. (Kamran et al., 2013) and Conboy et al. (Conboy and Conboy, 2009) and integrates best practices for prevention of observed complications in aging mouse experiments. We also provide guidance for longitudinal validation of blood chimerism among parabionts. This protocol can facilitate reproducible implementation of the surgical parabiosis model with limited technical challenges.

## Materials and equipment

### Animals

This protocol was developed using C57BL6 mice that were an average age of two-months-old (young [Y]) and 19-months-old (old [O]). Y wild-type C57BL6 and C57BL/6-Tg(CAG-EGFP)131Osb (Jackson Labs Stock: 006567) were used for GFP-blood chimerism experiments. YY, YO, and OO pairs were selected based on sex, age, and body weight and were either littermates or non-cage mates paired at the time of surgery. Mice were surgically paired for up to 5 weeks. Mice were housed in a facility with a 12/12-h light/dark cycle with *ad libitum* access to food and water.

### Reagents for surgical parabiosis

#### Antibiotics

− Enrofloxacin (100 mg/ml, Baytril 100, Bayer)
   • Dose: 2.5 mg/kg, subcutaneous (SQ) administration

#### Analgesia

− Carprofen (50 mg/ml, Rimadyl, Zoetis)
   • Dose: 5–10 mg/kg, SQ administration
− Buprenorphine SR-LAB (0.5 mg/ml, ZooPharm)
   • Dose: 0.5 mg/kg, SQ administration

#### Anesthesia

− Ketamine (K, 100 mg/ml, Ketaset, Zoetis)
   • Dose: 100 mg/kg (Table 1), intraperitoneal (IP) administration
− Xylazine (X, 100 mg/ml, XylaMed, VetOne)
   • Dose: 10 mg/kg (Table 1), IP administration
− Phosphate Buffered Saline (PBS, Gibco)
− Isoflurane (Iso, Fluriso, VetOne, 502017) (optional)
   • Inhalation administration at 2–3%

**TABLE 1:** Combination ketamine and xylazine (KX) anesthesia dosing recommendations for young and old C57BL6 mice prior to parabiosis surgery.

Weight (g)	Vol (ul) of KX 100mg/10mg//kg	Vol (ul) of KX 100mg/10mg//kg	Vol (ul) of KX 100mg/10mg//kg
	Y-Female	O-Female	Y- and O-Male
15–20	150	200	150
20–25	200	200	200
25–30	250	250	250
30–35	300	300	300
35–40	325	325	350
40–45	350	350	350

#### Perioperative

− Povidone-Iodine Swabstick 3’s (PDI, S41125)
− Alcohol swabs (BD, 326895)
− Optixcare Eye Lube (sterile)
− Collasate Postoperative Dressing (PRN Pharmacal)
− Collasate Silver (PRN Pharmacal)
− Triple Antibiotic Ointment (TAO)
   • Topical administration
− 0.9% NaCl (sterile saline)

#### Postoperative

− Napa Nectar (SE Lab Group)
− DietGel 76A (Clear H2O, 72-07-5022)
− Nutri-Cal (Vetoquinol)

### Reagents for confirming blood chimerism

− 0.5 M EDTA (Santa Cruz Biotechnology, sc-203932)
− Hanks Balanced Salt Solution 1X without phenol red (HBSS, Gibco, 1,417-095)
− Bovine Serum Albumin (BSA, Fisher BioReagents)
− VersaLyse Lysing Solution (Beckman Coulter, A09777)

### Equipment for surgical parabiosis

− 0.5 ml syringes with 27 G needles attached (BD, 305620)
− 1 ml syringes with 23 G needles
− 10 ml Sterile empty glass vial
− 2–3 heating pads
− Electric hair clippers (Wahl)
− Wrapped sterile gloves
− Polyline sterile fields (busse Hospital Disposables, 696)
− Surgical drapes (Haylard)
− Surgical drapes with rectangular holes cut in the center for surgical area exposure
− Cotton tipped swabs
− Autoclave
− Autoclavable static cages
− Autoclavable surgery pouches (5″x10″, chex-all II, 02401400)
− Autoclave tape
− Autoclavable instrument sterilization tray w/silicone mat (World Precision Instruments, 501728)
− Autoclavable toothed forceps (Fine Science Tools [FST],11053-10)
− Autoclavable curved, non-toothed forceps (Roboz, RS-101)
− Autoclavable surgery scissors (FST, 14568-09)
− 2–4 autoclavable hemostats (Kent Scientific)
− Surgical bonnet (Medchoice, 69909)
− Procedure mask (Haylard, EN 14683[BFE])
− Clean lab coat
− Micro bead research sterilizer (Benchmark Scientific, B1201)
− 3-0 non-absorbable, black braided, sutures (ETHICON, Perma-Hand Silk, K872H)
− 4-0 absorbable, violet braided, sutures (ETHICON, Coated Vicryl, J304H)
− Plastic weight boats or 1 ounce paper cups
− Alpha-dri bedding (Shepherd Specialty Papers)
− Iso-PADS (Otto Environmental)
− Two channel anesthesia stand (Kent Scientific, VetFlo-1215, optional)

### Equipment for confirming blood chimerism

− 1.5 ml Conical tubes
− 50 ml Falcon tubes
− FACS tubes
− 21 G or 23 G needles
− Gauze
− P100 or P200 pipette
− P100 or P200 pipette tips
− Microcentrifuge
− FACS tubes
− P1000 pipette
− P1000 pipette tips
− Attune NxT Acoustic Focusing Cytometer (4486516) (or alternative cytometer)
− FlowJo software
− Heating pad(s) and/or heat lamp

## Methods

### Parabiosis surgery

#### Preoperative

1) Weigh mice and select pairs. Ideally, parabiont body weights should not differ by more than 20%.
2) Using a 27 G needle, administer SQ injections of calculated doses of carprofen and enrofloxacin 12–24 h prior to surgery.
3) Prepare KX anesthesia at 100/10 mg//kg working dose (Table 1).
  • If using isoflurane as secondary anesthesia, prepare vaporizer.
4) Prepare one autoclaved surgical pack per pair of mice, containing one drape with a rectangular hole cut in the center and four to six cotton-tipped swabs.
5) Autoclave the instrument tray with lid, containing the surgical tools.
6) Autoclave static cages with iso-PADS or paper towel on the cage floor.

#### Surgical preparation

7) Move mice to the surgical room a minimum of 30 min prior to surgery for acclimation.
8) Set up recovery cages, consisting of autoclaved static cages with inserted iso-PADS, on a heating pad set to medium (approximately 35°C).
9) Prepare the sterile surgical area with a sterile field on top of a heating pad set to medium. Fold the sterile field and heating pad in half without touching the sterile field, to minimize hair transfer from clipping/shaving.
10) Administer KX anesthesia IP using a 27 G needle (Table 1).
  • Alternatively, isoflurane can be administered using a two-chamber vaporizer and/or a double nose cone. However, postoperative consciousness may occur more rapidly, relative to KX anesthesia.
11) As the mice become anesthetized, administer carprofen and enrofloxacin SQ using 27 G needles on the side opposite to the planned surgical side.
12) Apply Optixcare lubricant to both eyes of each mouse.
13) When no longer reflexive to toe pinch or eye touch and no whisker movement is visible, move the parabionts to the surgical prepping station. Place each mouse on their side, back-to-back on a heating pad set to medium that is covered with a blue surgical drape. This area should be separate from but adjacent to the surgical area. Shave fur from the elbow to the knee on the planned side of incision. The shave lines run approximately one to two cm above the elbow, to one to two cm below the knee. Clear all shaved fur from the ear to the rear foot.
  • Be careful to avoid nicking the skin. If the skin is nicked during shaving, clean the area with iodine and alcohol during the next step. The wound can be treated with topical Collasate or closed with 4-0 absorbable sutures at the end of surgery.
14) Once shaved, place mice back-to-back. Thoroughly clean the entire shaved areas, as well as the ears, tails, and feet, alternating between iodine and alcohol. Repeat three times.
15) Recheck mouse reflexes with toe pinch and eye touch; look for whisker movement. If responsive, wait for up to 15 minutes. If still responsive, a supplemental dose of K (25 ul for Y and 50 ul for O, 100 mg/kg) can be provided.
  • Alternatively, isoflurane can be administered at 2–3% using a vaporizer.
16) If eyes appear dry, re-apply Optixcare lubricant to both eyes of each mouse before moving to the sterile surgical area.
17) Move parabionts to the sterile surgical area, on a heating pad set to medium, and place them back-to-back with the shaved area facing up.
18) The anticipated duration of surgical preparation including anesthesia, shaving, and cleaning is approximately 30 min.

#### Surgery

**FIGURE 2:**
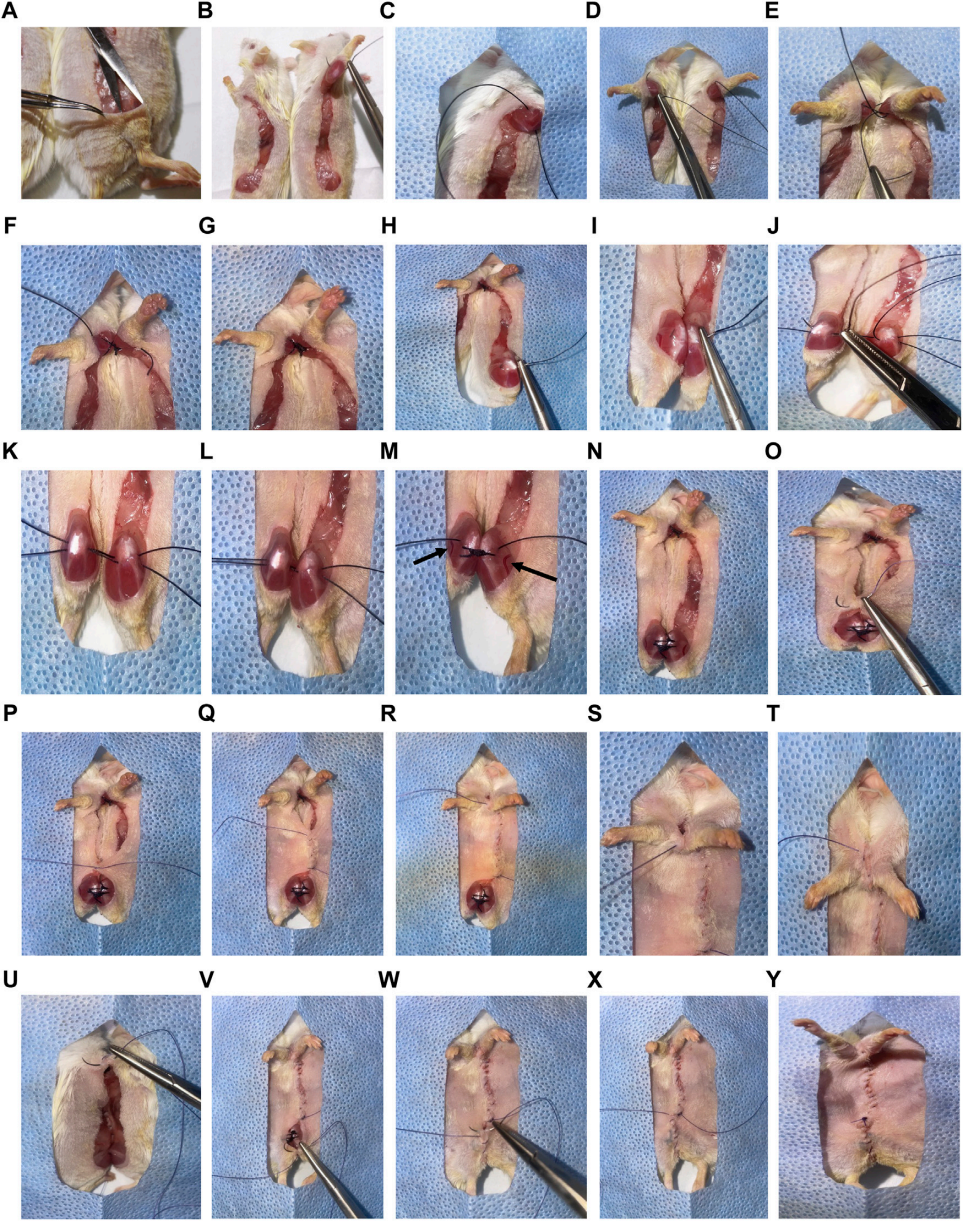
Overview of parabiosis surgery. **(A)** After generating the elbow to knee incision site, use non-toothed forceps to gently pinch and lift the top layer of skin at the knee joint area. Insert the tip of scissors underneath the skin and open the scissors, creating a pocket. **(B)** Use 3-0 non-absorbable sutures to attach the elbow of one parabiont to the elbow of the other parabiont. **(C)** Pass the needle above the olecranon joint by bending the elbow of the first mouse and pulling the suture through. **(D)** Bend the elbow of the second mouse, passing the same needle and suture under the elbow. Pull the suture through, leaving a small tail (5 cm). **(E)** Secure the joints and sutures with a surgeon’s knot, followed by two square knots. **(F)** Cut the ends short (less than 0.5 cm) leaving only sufficient length to prevent knot separation. **(F,G)** Repeat this step to create a second reinforcing ligature at the elbow. **(H)** Use 3-0 non-absorbable sutures to attach the knee of one parabiont to the knee of the other. Insert the needle medially under the knee joint and move the needle laterally under the joint. **(I–K)** Once the suture is pulled through one knee, continue to the other knee by inserting the needle laterally under the joint, and move the needle medially. Cut the suture leaving a 5 cm tail on each side with space between the knees. Repeat this again before tying the sutures. **(L)** Once the second suture is cut, pull the knees together. **(M)** Tie a surgeon’s knot, followed by two square knots. (Great saphenous vein and saphenous artery are indicated with arrows). **(N)** Repeat for both sutures. **(O)** Using toothed forceps and 4-0 absorbable sutures, connect the skin of the parabionts with a continuous suture. Start ventrally adjacent to the knee area. **(P)** Attach the skin with a surgeon’s knot, followed by two square knots, leaving a tail (5–10 cm) for final closure. **(Q–T)** Continue tight and closely positioned continuous sutures, moving cranially towards the elbows until unable to reach the skin on the dorsal side (beyond the elbows). **(U)** Lift the drape, and using scrubbed tails, feet, or ears, rotate mice into a prone position and continue tight, continuous suturing. **(V, W)** Once the knees are reached, flip the mice to a supine position, and continue suturing until the original knot is reached. **(X,Y)** Tie the continuous suture end to the initial 5–10 cm tail with a surgeon’s knot, followed by two square knots. Verify continuity of sutures. Perform single, interrupted sutures to close any openings or shaving-associated nicks. *For clear representation of surgical processes, albino (ICR) mice were used.

19) Unwrap the sterile sutures and autoclaved surgical packs over the sterile field allowing the items to drop into place. Set the autoclaved container with surgical tools immediately adjacent to the sterile field. Open the lid to the sterile tools, place the lid elsewhere. Discard current gloves.
20) If using the same surgical tools for more than one pair, turn on the hot bead sterilizer (280°C). Clean and sterilize tools between surgeries.
21) Don bonnet and surgical mask. Scrub hands with soap and water, and dry hands.
22) Open and carefully put on sterile gloves.
23) Retrieve autoclaved surgical tools and place them directly onto the sterile field.
24) Confirm lack of responsivity to toe pinch.
25) Using sterile, non-toothed forceps, superficially pull up the knee skin of one parabiont. Incise the skin longitudinally with sterile surgical scissors from 0.5 cm above the knee to 0.5 cm above the elbow joints. Carefully cut the connecting fascia creating “loose” skin along the incision lines. Trim small flaps of skin that are not flush with incision lines, especially at the joint areas. If using different sized animals, incise the smaller mouse dorsal to the midline and the larger mouse ventral to the midline (Figure 2B). This allows for better mobility following surgery and decreases dragging of the smaller mouse.
26) Using non-toothed forceps, gently pull up the skin at each joint area. Insert the scissors below the skin and open them to create a “pocket” of unattached skin (Figure 2A). This enables exposure for joint suturing.
27) Open and place the sterile drape with a pre-cut rectangular hole over connection areas of the mice.
28) Using 3–0 non-absorbable sutures, attach the elbow of one parabiont to the elbow of the other. Pass the needle above the olecranon joint by bending the elbow of the first mouse and pull the suture through (Figures 2B,C). Bend the elbow of the second mouse, passing the same needle and suture underneath the elbow (Figure 2D). Pull the suture through, leaving a small tail (5 cm). Secure with a surgeon’s knot, followed by two square knots (Figure 2E). Snip the ends short (less than 0.5 cm), leaving only sufficient length to prevent knot separation. Repeat this step to create a second reinforcing ligature at the elbow (Figures 2F,G). Bleeding in this area should be minimal, but if bleeding occurs, apply pressure using sterile cotton tipped swabs.
29) Using 3–0 non-absorbable sutures, attach the knee of one parabiont to the knee of the other. For the first mouse, insert the needle medially under the knee joint, and move the needle laterally (Figure 2H). Avoid lacerating the great saphenous vein and saphenous artery, which are located near this location (Figure 2M). Once the suture is pulled through one knee, continue to the other knee by inserting the needle laterally under the knee joint, and move the needle medially (Figure 2I). Cut the suture leaving about a 5 cm tail; also leave space between the knees (Figure 2J). Repeat this again, pulling the second suture through (Figure 2K). Once the second suture is cut, pull the knees together (Figure 2L). Tie a surgeon’s knot, followed by two square knots. Do this for both sutures (Figure 2M, N). As with the elbows, bleeding should be minimal, but if it occurs, apply pressure using sterile cotton tipped swabs.
30) Using toothed forceps and 4-0 absorbable sutures, connect the skin of the parabionts with a continuous suture. Start ventrally, adjacent to the knee area (Figure 2O). Attach the skin with a surgeon’s knot, followed by two square knots, leaving a tail (5–10 cm) for final closure (Figure 2P). Continue tight and closely positioned continuous sutures, moving cranially towards the elbows until unable to reach the skin on the dorsal side (beyond the elbows) (Figures 2Q–T).
31) Lift the drape, and using the scrubbed tails, feet and/or ears, rotate mice into a prone position. Return the drape with the fusion area exposed. Continue tight, continuous suturing moving caudally from the shoulder on the dorsal side (Figure 2U).
32) Once the knees are reached, flip the mice to a supine position, and continue suturing until the original knot is reached (Figures 2V–W).
33) Tie the continuous suture end to the initial 5–10 cm tail with a surgeon’s knot, followed by two square knots (Figures 2X,Y). Verify continuity of sutures. Perform single, interrupted sutures to close any openings or shaving-associated nicks.
34) Cover the ventral sutures with 1:1 TAO Collasate. Flip mice to a prone position and cover the dorsal sutures with 1:1 TAO Collasate.
35) Administer 0.5–1 ml warmed saline SQ to each mouse with a 27 G needle.
36) Using a 23 G needle, administer Buprenorphine SR SQ at the nape of the neck.
37) The surgery duration, from initial incision to completion of suturing should be 30 min or less. Extending beyond this time can lead to dehydration and/or increased risk of mortality.

#### Postoperative care

38) Move the paired mice to a sterile recovery cage containing an iso-PAD, placed on top of a heating pad set to medium. Monitor the mice hourly until they regain consciousness. If one parabiont regains consciousness significantly before the other, the conscious mouse can be administered 25–50 ul K, to reanesthetize. Once both mice have regained consciousness and are ambulatory, set the heating pad to low and adjust the cage so half of the cage floor is heated.
39) Provide Napa Nectar and DietGel on the recovery cage floor and recheck mice 4–6 h postoperatively. Leave the mice in the recovery cage in the procedure room overnight.
40) The next morning, administer carprofen analgesic and enrofloxacin antibiotic SQ, using a 27 G needle to each mouse.
41) If the mice are cool to the touch, administer warmed saline SQ using a 27G needle and keep on heat in the recovery cage until warm and ambulatory.
42) If both mice are alert and ambulatory, move the pair to a clean cage with Alpha-dri bedding. This bedding offers traction to assist with ambulation. Supplemental nesting and bedding options include nestlets and/or Cellu-nest. Provide Napa Nectar, DietGel, moist chow on the cage floor, and a new water bottle with a sipper tube.
43) Using 27 G needles, provide carprofen and enrofloxacin SQ, once daily for three days to each mouse.
44) Provide Napa Nectar, DietGel, and moist chow continuously throughout the study and monitor for evidence of intake.
45) Monitor mice daily (including weekends) until absorbable external sutures have dissolved. Absorbable skin sutures should not be forcefully removed. However, dissolving sutures can be trimmed to reduce mice from chewing and/or pulling on the sutures. If the skin appears completely healed and both ends of a suture section are exposed, use suture scissors to cut into smaller pieces, and remove with forceps. Sutures may begin breaking down as early as day seven but may not be weak enough to remove until days 10–14.
46) Once the sutures have dissolved, monitor mice daily on weekdays. Simultaneously euthanize parabionts within 5 weeks following surgery.

#### Handling of surgically paired mice

47) A “double scruff” technique is useful to gently and securely hold both parabionts when assessing ventral sutures.
48) Scoop the pair of mice with both hands and place on the feeder/on cage lid/etc.
49) Using one’s dominant hand, secure both tails.
50) Using the non-dominant hand, securely scruff one parabiont at the nape of the neck using the index finger and thumb.
51) Scruff the conjoined parabiont with the middle finger to gently pinch and push the skin of the nape onto the dorsal side of the index finger.
52) Once secure, lift and flip over the mice exposing their abdomens, securing their tails by wrapping them in between the ring and pinky fingers of the non-dominant hand.
53) Holding the parabionts in the palm of the hand or on the back of the hand is appropriate for assessment of dorsal sutures.

#### Surgical postoperative complications

**FIGURE 3:**
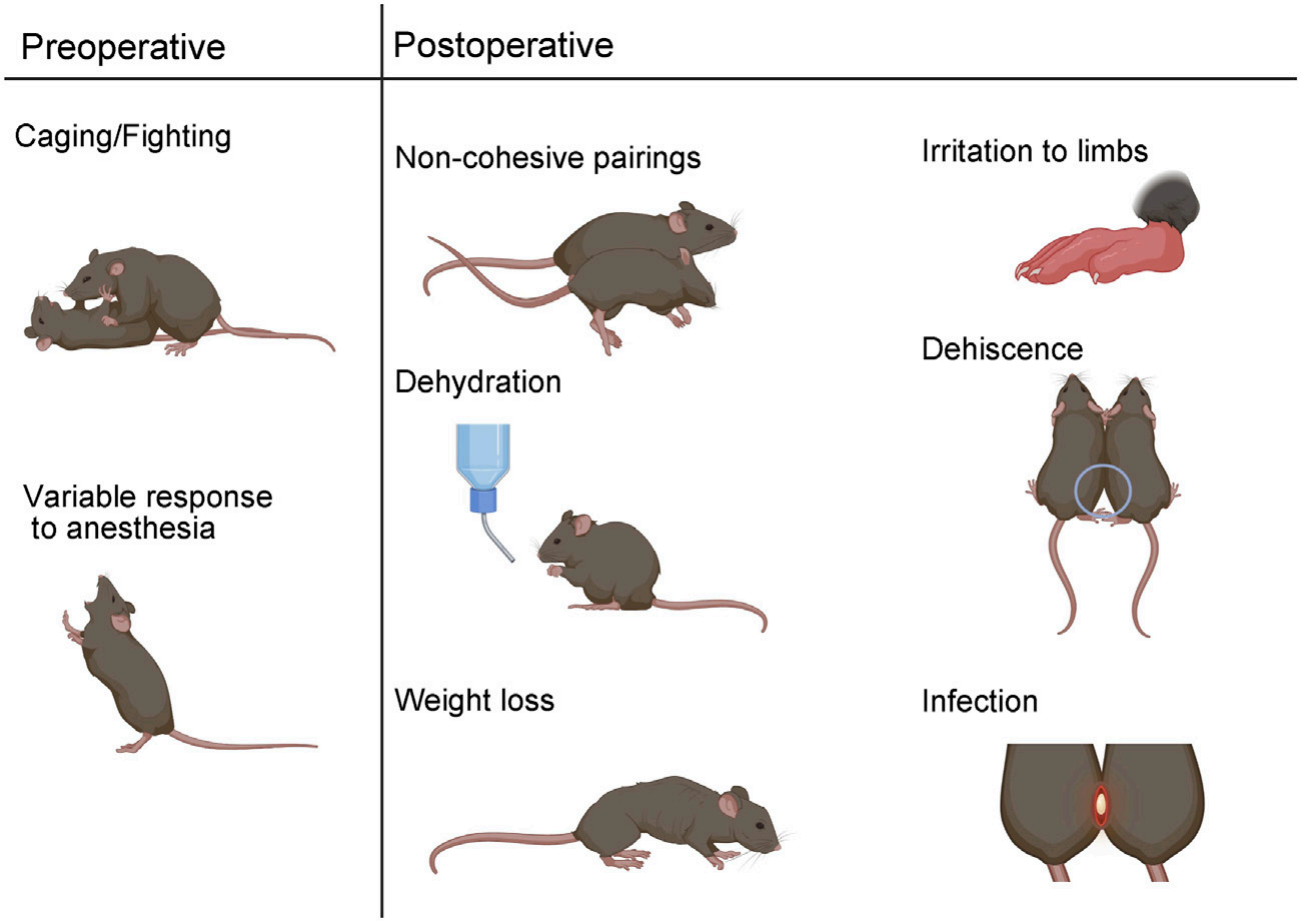
Surgical parabiosis complications. Preoperative complications can include aggressive behavior which can lead to non-cohesive unions. Variable response to anesthesia is another preoperative complication. Postoperatively, non-cohesive pairs can result from immune rejection or body weight differences greater than 20%. Dehydration, weight loss, irritation, dehiscence, and infection are additional adverse outcomes that can occur in the post-surgical phase.

**FIGURE 4:**
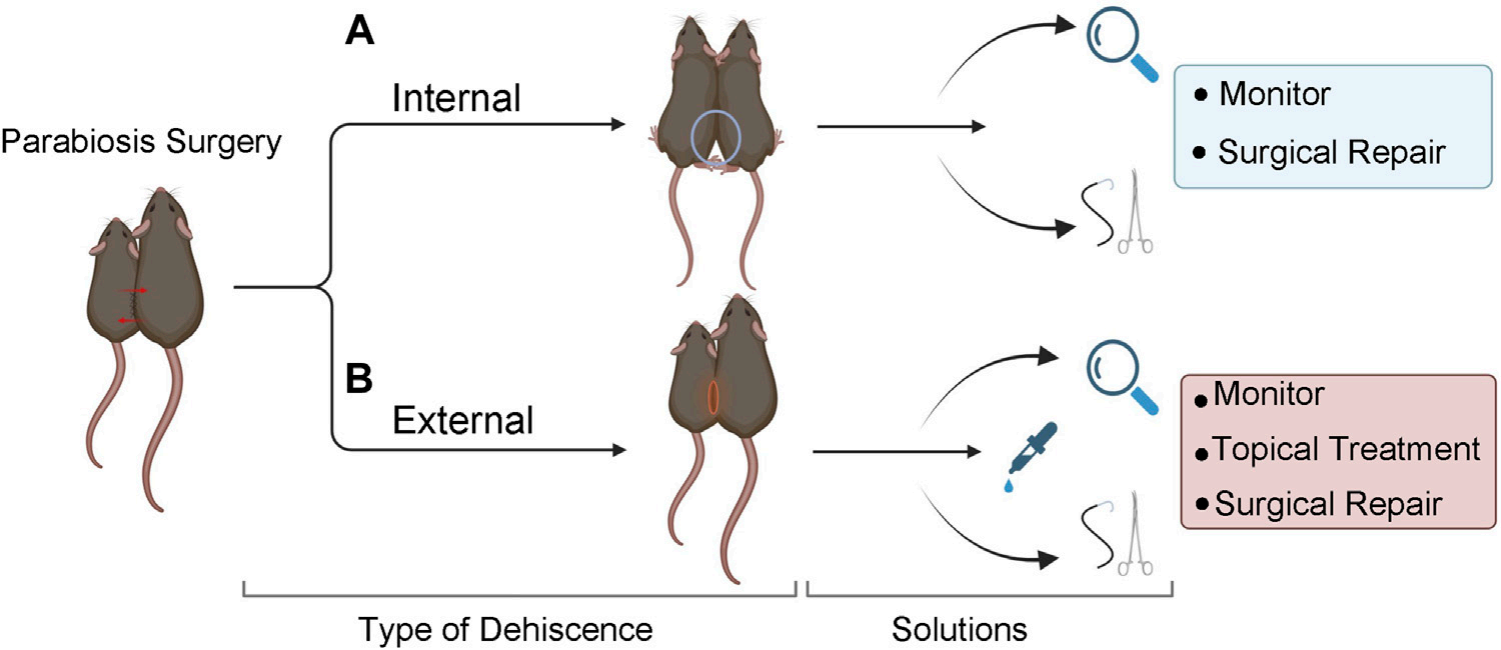
Solutions for internal and external dehiscence. **(A)** Internal dehiscence occurs when one or both joint unions have separated. Use a double knot technique at the time of surgery to prevent dehiscence. Surgically repair elbow separation. If knee separation occurs, observe or surgically repair, based on the severity and/or length of time after the original surgery. **(B)** External dehiscence occurs when the skin separates along the suture site. Treat with topical agents, monitor, and/or surgically repair. If both internal and external dehiscence occur at the same time, surgical repair is recommended.

**FIGURE 5:**
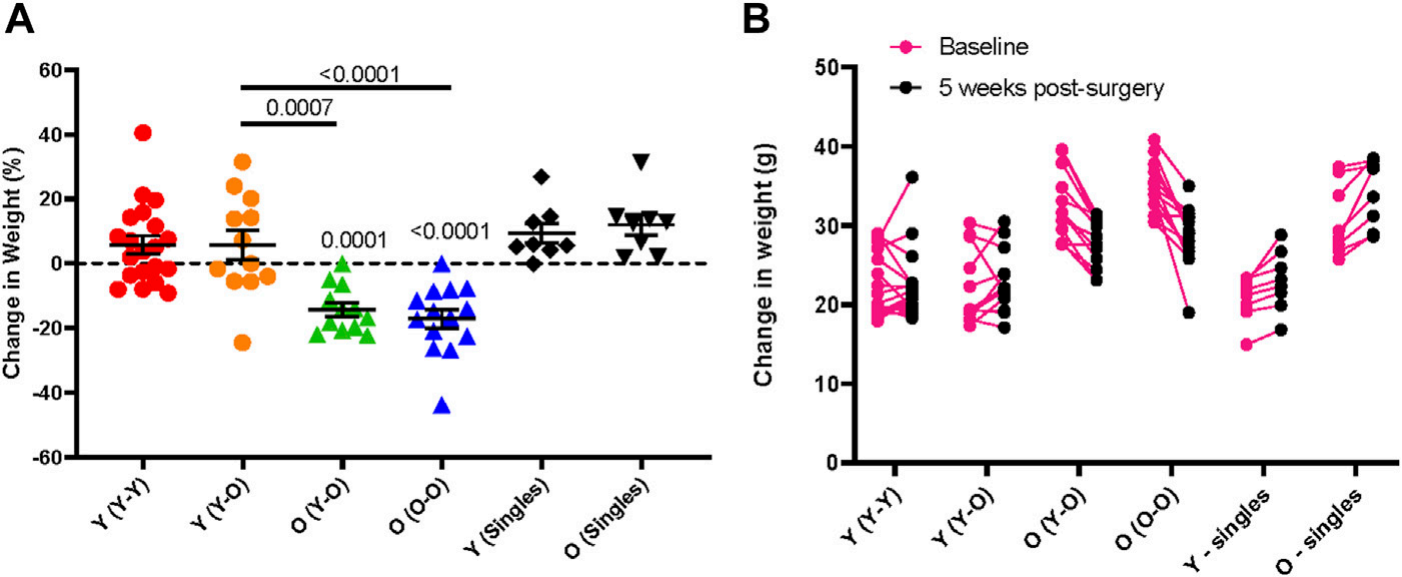
Age-dependent changes in body weight following parabiosis. Body weight change from baseline to 5-weeks following parabiosis surgery in females and males is depicted as **(A)** percentage change (Surgical pairs n = 6–10, singles n = 8; one-way ANOVA with multiple comparison testing) and **(B)** change in grams (g). Young mice maintain body weight and old mice lose weight after surgery.

54) Maintaining aseptic technique during surgery and immediately treating dehiscence (separation) can reduce the risk of infection. If infection occurs, administer enrofloxacin SQ to both mice once daily for a minimum of three days (Figure 3).
55) Irritation to the feet and/or limbs can result from improper surgical union of the joints. Surgical repair may be necessary. If irritation due to scratching occurs, trim nails and/or apply TAO.
56) If internal elbow dehiscence occurs, surgical repair is recommended to avoid other complications (Figure 4A).
57) If internal knee dehiscence occurs less than three weeks after surgery, surgical repair is advised. If internal knee dehiscence occurs greater than three weeks after surgery, close monitoring until the specified endpoint is recommended (Figure 4A).
58) If external dehiscence occurs at any point along the incision site, apply Collasate to the open area. Surgical repair is also an option (Figure 4B).
59) If signs of dehydration occur, such as tenting of the skin, administer warmed saline SQ.
60) Aged mice are at a greater risk of postoperative weight loss, relative to young mice (Figure 5). Weigh mice prior to surgery, and weigh pairs weekly. If a decline in body weight is observed, offer Nutri-Cal, in addition to DietGel and moist chow.

### Confirmation of blood chimerism following parabiotic surgery

**FIGURE 6:**
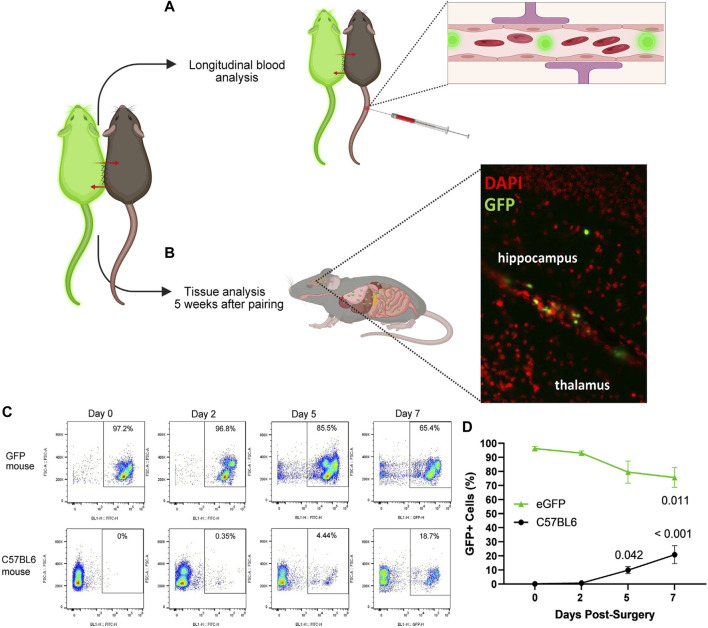
Strategies for confirming blood chimerism following parabiotic surgery. Using a transgenic model that expresses a fluorescent reporter in blood cells, such as eGFP, and C57BL6 heterogenic pairs, circulatory exchange can be confirmed by **(A,C,D)** longitudinal blood analysis and/or **(B)** tissue analysis after 5 weeks of pairing, demonstrating GFP + cells in the brain of a C57BL6 parabiont using fluorescent microscopy. **(C)** Representative flow cytometry in eGFP (top row) and C57BL6 (bottom row) heterogenic parabionts at 0, 2, 5, and 7 days postoperatively. **(D)** Abundance of GFP + cells in blood circulation in eGFP (green triangles) and C57BL6 (black circles) heterogenic parabionts, with conclusive blood exchange as early as day 5. (Friedman test with multiple comparisons, compared to day 0. Mean ± SEM, *n* = 6 mice per genotype.)

The following protocol enables flow cytometric detection and quantification of GFP-positive (+) cells in blood samples following surgical parabiosis of heterogenic pairs (Figure 1), comprised of wild-type C57BL6 and eGFP mice. The following steps should be conducted on blood from each parabiont prior to surgery (baseline) and longitudinally following surgery. Blood from non-paired C57BL6 and eGFP mice can be used as negative and positive controls, respectively. Analyses at additional timepoints can reveal the time course of parabiotic blood exchange (Figure 6).

#### Blood collection and processing

1) Label 1.5 ml conical tubes with identification number for each parabiont or control sample.
2) Prepare 10 mM EDTA in HBSS. For 50 ml solution, combine 1 ml 0.5 M EDTA with 49 ml HBSS in a 50 ml Falcon tube.
3) Aliquot 300 uL EDTA/HBSS to each labeled conical tube.
4) Select a pair and place them in a cage on a heating pad set to medium or use a heat lamp. Warming the mice dilates the veins, which increases blood flow and renders the veins easier to identify.
5) Scoop the mice, place them in the cage feeder, and gently slide the tails through the slits. This will allow movement while securing their rear ends against the lip of the feeder.
6) Gently grip the base of the tail with the forefinger and thumb. Locate a lateral tail vein, and gently puncture the tail vein with a 23 G needle. A blood drop should appear.
7) Using the forefinger and thumb, stroke the mouse’s tail caudally from the base to encourage blood to pool at the puncture site.
8) Using a P100 or P200 pipette, draw up 20 ul 10 mM EDTA into the pipette tip, and apply the end of the pipette tip to the blood drop. Blood will move into the tip via capillary action.
9) Collect 30–40 ul of blood and transfer to a 1.5 ml conical tube.
10) Apply gentle pressure to the puncture wound with gauze until bleeding stops.
11) Repeat steps 6–10 for all control and experimental mice.
12) Centrifuge the blood at 400 g, 20°C for 4 min.
13) Remove and discard the supernatant.
14) Add 100 ul 1% BSA-FACS buffer to the cell pellet and vortex until the pellet is thoroughly resuspended.
15) Add 1 ml Versa-Lyse solution to identifier-labeled FACS tubes.
16) Transfer the cell suspension to a FACS tube and allow the sample to incubate at room temperature for 10 min.
17) Analyze blood using a using an Attune NxT Acoustic Focusing cytometer. Analyze the samples at 1,000 ul/min, up to 10,000 events per second.
18) Save the results as an FCS file for analysis using FlowJo software.

#### Cytometry analysis

19) Open FlowJo software.
20) Click and drag FCS files into the FlowJo workspace.
21) Select the FCS file corresponding to the non-paired C57BL6 negative control sample. Draw a gate on the forward scatter (FSC) versus side scatter (SSC) plot that excludes debris. This is the “live cells” gate.
22) Using the population in the “live cells” gate, change the axes to forward scatter area (FSC: A) vs. forward scatter height (FSC: H). Draw a diagonal gate from the bottom left to the top right of the plot that includes all cells with approximately 1:1 ratio of FSC:A to FSC: H. This is the “single cells” gate.
23) Using the population in the “single cells” gate, change the axes to FSC versus GFP. Draw a gate that selects the GFP+ population and excludes the negative population. This is the “GFP+ gate”. For a C57BL6 mouse at baseline, this gate may contain a few events due to background fluorescence but should not exceed 0.50% of events inside the “single cells” gate.
24) Complete steps 1–5 for an eGFP control sample; all GFP+ cells should be inside the true positive gate.
25) Apply the gates to all samples by clicking and dragging the gates onto them. Check each sample individually to ensure accurate gating.
26) Click “Table Editor” in the FlowJo workspace. Click and drag the “GFP+ gate” from one of the samples into the table editor. Select “Create Table” to produce percentages of GFP+ cells for each sample.
27) Correct for background fluorescence by subtracting the percentage of GFP+ cells identified in control C57BL6 sample(s) from the percentage of GFP+ cells identified in each experimental sample.
28) Perform statistical tests and graph results as appropriate.

## Results

### Parabiosis surgical model complications and solutions

Complications that manifest during the preoperative, operative, or postoperative phase of parabiosis experimentation can compromise hypothesis testing (Conboy et al., 2013). Anticipated complications include parabiotic disharmony, dehiscence, dehydration, weight loss, skin irritation, infection, and death (Figure 3).

Careful consideration of pairing in the experimental design phase can increase the likelihood of successful unions. Parabiotic disharmony has historically been used to describe immune rejection and can be identified by severe lethargy and/or death of a parabiont (Dragstedt and Cooper, 1923; Conboy et al., 2013). Pairing mice of the same or similar genetic background can reduce the risk of parabiotic disharmony (Conboy et al., 2013). Non-cohesive pairing is another form of disharmony that can result from a significant mismatch in weight or in cases of aggressive behavior (Yang et al., 2021). We recommend pairing mice with no more than a 20% difference in total body weight. Since male mice can exhibit aggressive behavior, it is not recommended to co-house mice for habituation prior to surgery. Rather, males can be surgically paired with long-term cage mates or with a naïve parabiont at the time of surgery. Aggressive behaviors can still occur postoperatively and should be closely monitored.

Sensitivity to anesthesia is an important consideration for any survival procedure and particularly for parabiosis surgery, since two mice must be simultaneously maintained under sedation. The time it takes to reach the correct plane of anesthesia and the duration in that plane can vary by sex, weight, age, and strain (Levin-Arama et al., 2016). Accordingly, anesthesia must be titrated according to experimental parameters. We offer an optimized dilution chart for combination KX anesthesia based on sex, weight, and age for C57BL6 mice (Table 1). In our hands, females require more sensitive titration based on age and weight, relative to males. During surgery, it is critical to monitor breathing rhythm and reflexes to maintain an adequate plane of anesthesia. Supplementary K or isoflurane can be applied as secondary forms of maintenance anesthesia. Xylazine is not recommended for maintenance anesthesia, since prolonged use can lead to central nervous system depression resulting in difficulty maintaining an adequate respiratory rate (Jaber et al., 2014; Levin-Arama et al., 2016).

Dehiscence can occur internally at the joints and/or externally at the skin incision site (Figure 4). External dehiscence can occur while the incision sutures are healing. To promote incision site healing, apply TAO mixed with Collasate (1:1) to the entirety of the incision line at the end of surgery. If external dehiscence occurs, Collasate or Collasate Silver can be applied topically to encourage healing of the separated skin. Dehiscence can cause an opening to the body cavity that increases the risk of infection. If dehiscence-related signs of infection occur, including reddening and inflammation of the skin or discharge, administer enrofloxacin SQ, once daily for a minimum of three days. If external dehiscence is moderate to severe, repair surgically under anesthesia. To prevent internal dehiscence, we recommend attaching the joints using two surgeon’s knots, each followed by two square knots as described in the protocol (Figures 2F,G,M,N). Internal separation of the knees may be less deleterious, relative to elbow dehiscence, particularly if knee dehiscence occurs multiple weeks following surgical union. Internal separation of the elbows can result in twisting, which can lead to constriction of the skin and vasculature, as well as dehydration. Surgical repair of elbow dehiscence is highly recommended. If both knee and elbow joint dehiscence occurs, surgical repair is required. The present experimental design of parabiotic union of up to five weeks was based on prior studies demonstrating this duration is sufficient to transfer progeronic or rejuvenative outcomes, to young or old counterparts, respectively (Conboy et al., 2005b; Villeda et al., 2011; Villeda et al., 2014; Liu et al., 2017). We acknowledge that shorter (Ruckh et al., 2012) or longer (Kiss et al., 2020; Yousefzadeh et al., 2020) unions may be sufficient or required for specific experimental questions, but longer-term unions may increase risk for adverse events, including dehiscence.

Other potential postoperative complications include dehydration and significant weight loss. Thorough daily observations and administration of supplements or treatments can prevent or partially mitigate these issues. Subcutaneous delivery of antibiotic is preferred over drinking water administration, as the latter can lead to inadequate voluntary water consumption and subsequent dehydration. To promote fluid and food intake, we recommend offering Napa Nectar, DietGel, and moist chow on the cage floor during the week following surgery; these supplements can be given for the entire study. Longitudinal change in body weight of isochronic and heterochronic young and old parabionts demonstrates that young parabionts maintain body weight over a five week parabiosis study, but old mice lose significant body weight. These outcomes are consistent in both isochronic and heterochronic pairs (Figure 5). Thus, old mice are particularly susceptible to body weight loss following parabiosis surgery.

Skin irritation can occur adjacent to suture sites. Externally, this can result from scratching and can be treated by applying TAO and performing nail trims. Swelling, irritation, or dragging of limbs can also occur, which can result from internal knots that are too tight or sub-optimally located. In our hands, this complication arises more frequently in males. This can be treated with TAO and/or carprofen, but if swelling and irritation does not resolve within five days, surgical repair is recommended. To minimize technical challenges, we highly recommend implementation of a carefully designed pilot study prior to experimental scaling and consultation with vivarium veterinary staff when unexpected complications arise.

### Confirmation of blood chimerism by flow cytometry

Confirmation of circulatory exchange following surgical parabiosis can be confirmed through blood or tissue analyses (Figure 6). Here, we leveraged flow cytometry to confirm the time course of chimerism among Tg(act-EGFP)Y01Osb (eGFP) and C57BL6 heterogenic parabionts. From the tail vein, blood samples were longitudinally collected at baseline prior to surgery and two, five, and seven days following surgical union (Figure 6C). Erythrocytes were lysed and flow cytometry was performed to quantify the percentage of GFP+ cells in C57BL6 and eGFP parabiont blood samples. At baseline and two days after parabiosis surgery, GFP+ cells were not reliably detected above background in C57BL6 parabionts joined to eGFP parabionts. Blood samples collected at day five demonstrated evidence of blood chimerism, with C57BL6 parabiont blood cell samples comprised of an average of 9.9% GFP+ cells. By day seven, an average of 20.9% C57BL6 parabiont blood cells were GFP+ cells (Figure 6D). Based on these data, conclusive circulatory exchange can occur for approximately four weeks within a five week study.

## Discussion

Surgical parabiosis is a unique and powerful model useful for discovery of circulatory mechanisms that mediate age-related dysfunction and conversely, mechanisms responsible for youth-related rejuvenation. From a technical perspective, parabiotic surgery presents many challenges. Through careful design and implementation of a standardized protocol, the model can be reproducibly implemented. Both preventative and reactive measures discussed herein are important for ensuring animal welfare and reducing the risk of adverse outcomes that can compromise study execution.

We and many others view the parabiosis model as an important tool to study hallmarks of aging (Lopez-Otin et al., 2013). For example, parabiosis has been used to demonstrate the effect of circulating factors on stem cell-related regenerative capacity (Conboy et al., 2005a; Katsimpardi et al., 2014; Sinha et al., 2014; Ma et al., 2022). Parabiotic exchange has also been used to demonstrate the capacity of young blood to reduce markers of cellular senescence in an old heterochronic parabiont, as well as increase markers of cellular senescence in a young heterochronic parabiont (Yousefzadeh et al., 2015). In the present report, we highlight specific challenges for which aged mice may experience greater risk, such as weight loss. Age-related sensitivity to anesthesia is another important experimental consideration. Overall survival following parabiosis surgery has been previously estimated at approximately 75% (Villeda et al., 2011), and we have observed that mouse strain, sex, and age can influence survival within defined experimental groups. Implementation of our optimized protocol can increase surgery success and reduce complications to facilitate important ongoing research focused on aging and regeneration. Moreover, many of the experimental complications and solutions discussed here may be useful for other types of parabiotic experimentation, including pharmacology, immunology, neurology, endocrinology, and other biomedical fields.

Our protocol includes a flow cytometry-based method for model validation and/or tracking blood chimerism over a time course. This method requires the heterogenic union of a wild-type parabiont and a transgenic parabiont harboring a fluorescent reporter within blood cells. Using this approach, we confirmed that blood circulation is reliably shared as early as five days following surgery. This is relatively consistent with prior studies, including reported sharing of blood by seven days post-surgery based on tracking of intravenous Evans blue dye (Donsky and Goldschneider, 1992) or GFP+ blood cells in a wild-type parabiont (Castellano et al., 2016). Similarly, using heterogenic pairing of transgenic mice harboring CD45.1 and CD45.2 cells, Wright et al. detected cross-circulation as early as three days after surgery (Wright et al., 2001).

In summary, this optimized protocol can enhance feasibility for reliably implementing the gold-standard parabiosis surgical model. This method has been optimized specifically to minimize surgical complications and achieve experimental end goals for the purpose of investigating longitudinal effects of surgical pairing of mice for aging studies.

## Data Availability

The original contributions presented in the study are included in the article, further inquiries can be directed to the corresponding author.
